# High-voltage pulsed radiofrequency improves ultrastructure of DRG and enhances spinal microglial autophagy to ameliorate neuropathic pain induced by SNI

**DOI:** 10.1038/s41598-024-55095-5

**Published:** 2024-02-24

**Authors:** Ri Chen, Xueru Xu, Youfen Yu, Yanqin Chen, Chun Lin, Rongguo Liu

**Affiliations:** 1 Department of Pain Management, Fujian Provincial Hospital, Shengli Clinical Medical College of Fujian Medical University, Fuzhou, Fujian China; 2 Department of Anesthesiology, Women’s and Children’s Hospital of Xiamen University, Xiamen, China; 3 School of Basic Medical Sciences, Institute of Pain Research, Fujian Medical University, Fuzhou, Fujian China

**Keywords:** HVPRF, Neuropathic pain, Autophagy, ATF3, TNF-α, IL-10, Cytokines, Neuroimmunology

## Abstract

Neuropathic pain (NeP) is intractable for which many therapies are ineffective. High-voltage pulsed radiofrequency (HVPRF) on dorsal root ganglion (DRG) is considered an effective treatment for NeP. The aim of this study is to explore the therapeutic voltage for the optimal efficacy of PRF and the underlying mechanisms. The radiofrequency electrode was placed close to the L5 DRG of rats with spared nerve injury (SNI) and emitted current by the corresponding voltage in different groups. Four different voltages (45 V, 65 V, 85 V, and 100 V) of PRF on DRG significantly alleviated the SNI-induced NeP, reduced the levels of activating transcription factor 3 (ATF3) in DRG, improved the ultrastructure of DRG, and promoted autophagy in spinal microglia to varying degrees and partially reversed the increased expression of TNF-α and the reduced expression of IL-10 in spinal cord dorsal horn (SCDH). The beneficial effect of 85V-PRF was superior to those of other three PRF treatments. The underlying mechanisms may be related to repairing the DRG damage and improving the DRG ultrastructure while regulating spinal microglial autophagy and thereby alleviating neuroinflammation.

## Introduction

Neuropathic pain (NeP) is a kind of intractable pain defined by the International Association for the Study of Pain as a pain arising after a lesion or disease affecting the somatosensory system^1^. Due to its high incidence (6.9–10% of the general population)^2^, complex pathogenesis and heavy disease burden, NeP is an urgent medical challenge calling for efficient treatments^3^. However, conventional pharmacological or surgical treatments often fail to meet treatment needs due to adverse side effects, multiple complications and complex operations.

Pulsed radiofrequency (PRF) is a minimally invasive technology proposed by Sluijter^2,4^ and has been widely used to manage several types of refractory chronic pain, such as postherpetic neuralgia^5^, cervicogenic headache^6^ and trigeminal neuralgia^7^. This technique works by delivering electrical fields and thermal pulses through the catheter tip to the target nerve or tissue without damaging these structures, and the clinical standard PRF parameters are as follows: the output voltage is 45 V, the current action time is 20 ms, the intermittent period is 480 ms, the oscillating frequency of the alternating current is 500 kHz and the maximum temperature of the electrode does not exceed 42 °C^8^. However, the effect of PRF followed the standard voltage (45 V) lasts only 4.5–6 months, and some patients need to undergo the procedure several times^9,10^. To ensure satisfactory efficacy, further research is needed to confirm the optimal treatment parameters, such as the output voltage and duration. In recent years, scholars have conducted clinical investigations to identify more effective PRF modalities and revealed that the high voltage output can improve the therapeutic efficacy of PRF. A previous study revealed that high-voltage PRF (HVPRF) in NeP patients induces a considerable therapeutic effect on short-term pain relief and substantial improvements in the quality of life^11^. Another study evaluated the efficacy of HVPRF (76.50 ± 5.61 V) in comparison with standard-voltage PRF (SVPRF, 45 V) for the treatment of acute herpes zoster neuralgia. The HVPRF can rapidly and steadily reduce the pain degree, improve the sleep quality, reduce the doses of anticonvulsants and analgesics, and decrease the incidence of clinically meaningful postherpetic neuralgia^12^. Our previous animal studies have demonstrated that 85 V PRF produces a better analgesic effect than 45 V PRF applied to the dorsal root ganglion (DRG) in spared nerve injury (SNI) rats^13^. These results indicated that the increase in output voltage might result in a greater reduction in NeP. Nevertheless, the optimal clinical voltage for maximum therapeutic effect and the underlying mechanisms of the advantage of HVPRF over SVPRF remain to be elucidated.

Recent research into the mechanisms underlying NeP has been increasingly focused on the regulation of inflammatory homeostasis. Microglia are considered the primary immune cells in the central nervous system, and their function and status are determining factors in the progression of NeP^14–16^. It has been revealed that autophagy is involved in the development of neurodegenerative diseases by regulating neuroinflammation mediated by microglia in the spinal cord^17,18^. A recent study confirmed that the autophagy-lysosomal pathway participates in chronical constriction injury (CCI)-induced NeP in rats^19^. Another study claimed that activation of neuronal autophagy might be a strategy for the treatment of SNI-induced NeP^17^. Previous studies have proven that appropriate autophagic modulation can alleviate pain behavior by enhancing the anti-inflammatory response in an SNI rat model^20^. These studies all suggested that the dysregulation of autophagy underlies NeP. However, whether PRF alleviates NeP by modulating the inflammatory response via autophagy remains obscure.

Thus, the aim of this study was to explore the optimal voltage for the therapeutic effect by comparing the pain reduction produced by four voltages of PRF applied to the DRG in SNI rats. In addition, we analyzed the changes in DRG ultrastructure, activating transcription factor 3 (ATF3) expression in the DRG, autophagy in microglia, and the expression levels of TNF-α and IL-10 in the spinal cord dorsal horn (SCDH) to provide a reliable basis for understanding the potential mechanisms of the HVPRF-induced NeP relief.

## Results

### HVPRF applied to the L5 DRG produced a remarkable reduction of NeP in SNI rats

**Figure 1:**
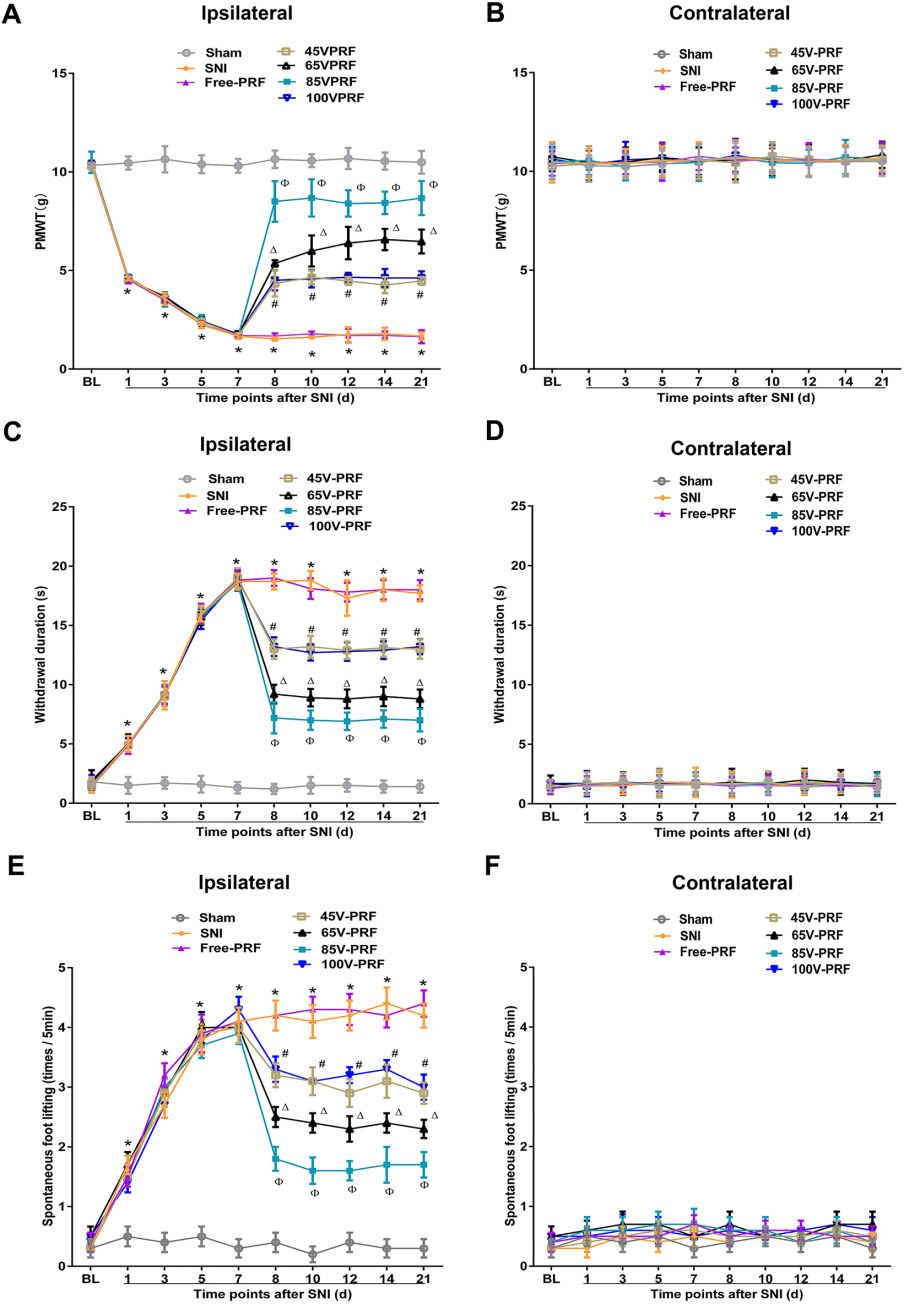
Single PRF applied on DRG alleviated SNI-induced NeP. The PMWTs **(A)**, the withdrawal duration of cold plate test **(C)**, and the times of SFL **(E)** were partially alleviated in the four treatment groups after PRF and maintained throughout the experiment compared to those in the SNI group. Furthermore, no significant difference in these pain behaviors were observed between the 45VPRF and the 100VPRF groups. In comparison, the PMWTs, the withdrawal duration of cold plate test, and the times of SFL were relieved in the 65VPRF group at all time points after the 8th day of SNI, which were better than those in the 45VPRF and 100VPRF groups, but were significantly worse than those in the 85VPRF group. Changes in PMWTs **(B)**, the withdrawal duration of cold plate test **(D)**, and the times of SFL **(F)** in the contralateral hind paw in all groups within 21 days post-SNI. One-way ANOVAs followed by the Tukey’s multiple comparisons test, n = 10 for each group. Data are expressed as the mean ± SD. *P < 0.05 vs. Sham; ^#^*P* < 0.05 vs. SNI; ^∆^*P* < 0.05 vs. 45VPRF; ^Φ^*P* < 0.05vs. 65VPRF.

To investigate the therapeutic effects of HVPRF on the pain behavior induced by SNI, we primarily evaluated three sensory behavioral functions affected by pain. Sensory function was tested by assessing the mechanical allodynia (paw mechanical withdrawal threshold, PMWT), cold allodynia (withdrawal duration of cold plate test), and spontaneous pain (spontaneous foot lifting, SFL) (Fig. 1). The baselines of these pain behaviors were not significantly different among all groups (Fig. 1A,C,E, all *P* > 0.05). A two-way repeated-measures ANOVA revealed that there were significant group effect, time effect, and group × time interaction in the three pain behaviors, respectively (numerical values of the statistics were shown in Supplementary Table S1). The three pain behaviors were observed on the 3rd day and peaked on the 7th day post-SNI. Thereafter, stable levels of these pain behaviors were maintained throughout the whole experiment. There were significant differences in these pain behaviors between the SNI and the Sham groups (Fig. 1A,C,E all *P* < 0.05). No significant difference in these pain behaviors were observed between the SNI and the Free-PRF groups (Fig. 1A,C,E, P > 0.05). No significant change in these pain behaviors were observed in the contralateral hind paw (Fig. 1B,D,F, all *P* > 0.05).

The PMWTs, the withdrawal duration of cold plate test, and the times of SFL were partially alleviated in the four treatment groups after PRF and maintained throughout the experiment compared to those in the SNI group (Fig. 1A,C,E all *P* < 0.05). Furthermore, no significant difference in these pain behaviors were observed between the 45VPRF and the 100VPRF groups (all *P* > 0.05). In comparison, the PMWTs, the withdrawal duration of cold plate test, and the times of SFL were relieved in the 65VPRF group at all time points after the 8th day of SNI, which were better than those in the 45VPRF and 100VPRF groups (all *P* < 0.05), but were significantly worse than those in the 85VPRF group (all* P* < 0.05). These results revealed that the analgesic efficacy of HVPRF was superior to that of SVPRF for NeP.

### HVPRF therapy achieved better improvement of the L5 DRG ultrastructure and less neuronal damage than SVPRF in rats with SNI

The ultrastructure and function of DRG neurons are interconnected. We used transmission electron microscope to observe the effect of PRF on the ultrastructure of the DRG. The results showed that the structure of the left L5 DRG neuronal soma were regular with homogeneous cytoplasm and uniform and loose chromatin in the Sham group. The nerve fibers were arranged in concentric circles with distinct myelin sheath layers. In the SNI group and Free-PRF group, the soma and myelin sheath of the DRG were severely damaged with nerve demyelination on the 14th and 21st day after SNI. Specifically, the neuronal soma displayed cytoplasmic shrinkage or swelling, nuclear pyknosis and displacement, and heterochromatin aggregation, and the membrane of cells was obscure with slightly enlarged space. The nerve medullary sheath had an unclear layer structure characterized by axis cylinder destruction and even exhibited a honeycomb-like appearance and fractured loose layers.

**Figure 2:**
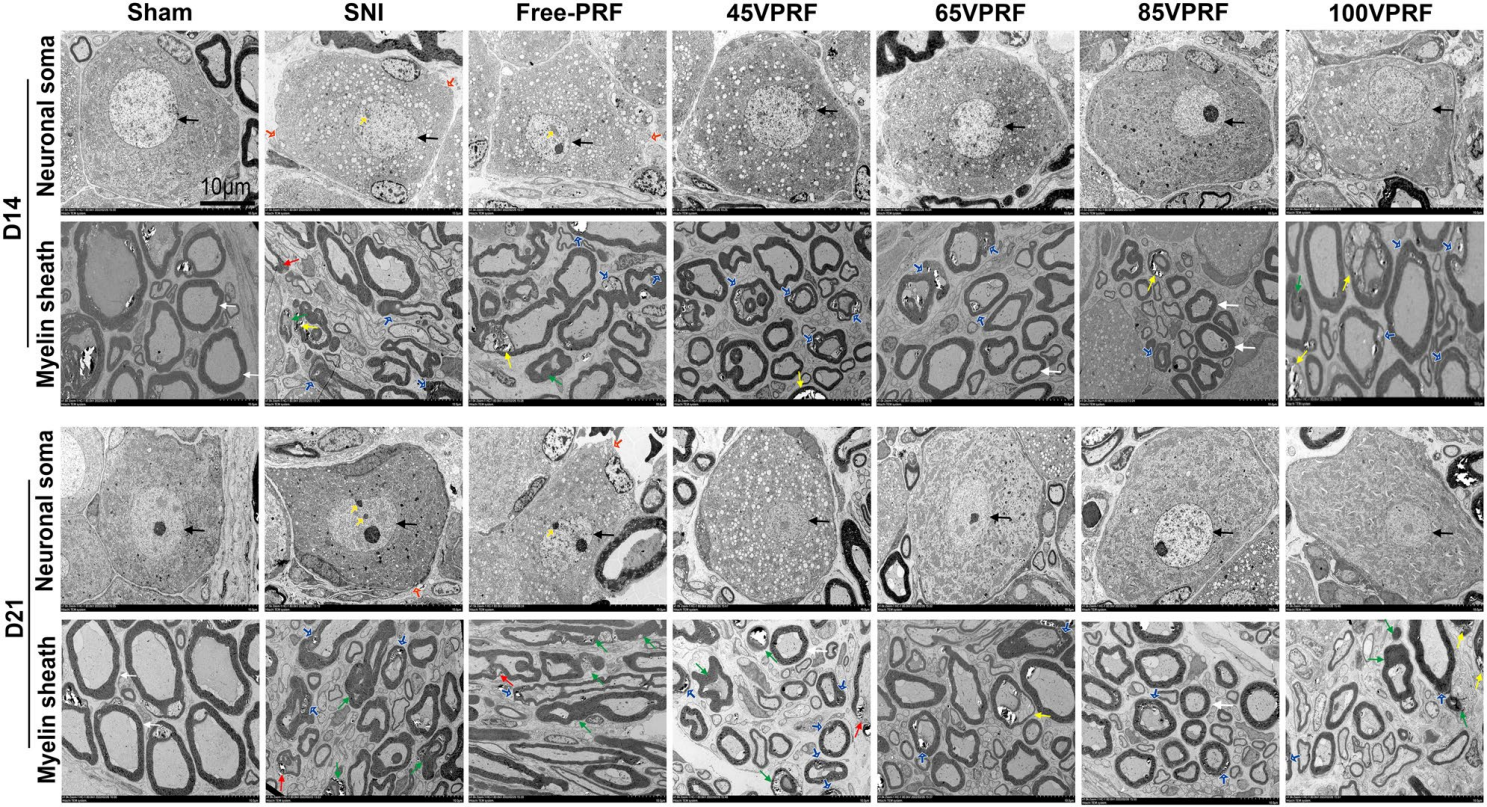
The ultrastructural changes in the left L5 DRG on the 14th and 21st days after SNI in different groups by transmission electron microscopy. The morphology of neuronal somata and myelin sheaths of L5 DRG neurons on days 14 and 21 after SNI in all groups. Neuronal somata: neuron nucleus (black arrow), the heterochromatin aggregation (yellow arrow), and the membrane of cells was obscure with slightly enlarged space (red arrow). Myelin sheath: normal (white arrow), separation in myelin configuration (blue arrow), interruption in myelin configuration (red arrow), honeycomb appearance (yellow arrow), collapsed myelin-forming ovoids (green arrow). Scale bar = 10 μm; magnification × 1000.

The structure of the DRG neuronal soma in four PRF groups were slightly abnormal, which showed that the nuclear pyknosis and displacement, and heterochromatin were lighter than those in the SNI and Free-PRF groups (Fig. 2).

Table 1 showed the grades of ultrastructure of myelinated axons in each group. The grades in the four treatment groups were better than those in the SNI and Free-PRF groups, which showed the worst grades (Tables 1, 2, all *P* < 0.05). No significant difference was found between the 45VPRF and 100VPRF groups (*P* > 0.05). Moreover, the grades in the 65VPRF group were better than those in the 45VPRF and 100VPRF groups (all *P* < 0.05), but were significantly worse than those in the 85VPRF group (all* P* < 0.05). Among all PRF treatment groups, the grades of ultrastructure were best in the 85VPRF group (Tables 1, 2, all *P* < 0.05).

**Table 1:** Semi-quantitative ultrastructural grading scores of myelinated axons in all groups on the 14th and 21st days after SNI.

Grade	Sham		SNI		Free-PRF		45VPRF		65VPRF		85VPRF		100VPRF	
	D14	D21	D14	D21	D14	D21	D14	D21	D14	D21	D14	D21	D14	D21
Grade0	227	230	34	29	31	27	59	62	71	73	102	99	57	61
Grade1	23	20	30	32	32	35	50	44	83	78	96	96	51	46
Grade2	0	0	41	50	38	37	35	41	38	45	26	28	39	42
Grade3	0	0	48	44	54	59	43	42	26	23	15	18	34	36
Grade4	0	0	97	95	95	92	63	61	32	31	11	9	69	65

*PRF* pulsed radiofrequency, *Free-PRF* free pulsed radiofrequency, *SNI* spared nerve injury.

**Table 2:** Statistical analysis of ultrastructural grading data of myelinated axons on the 14th and 21st days after SNI.

Pairwise comparisons	D14		D21	
	*H*	*P*	*H*	*P*
Sham—85VPRF	− 4.665	0.000	− 4.928	0.000
Sham—65VPRF	− 8.612	0.000	− 8.871	0.000
Sham—45VPRF	− 12.650	0.000	− 12.903	0.000
Sham—100VPRF	− 12.739	0.000	− 12.814	0.000
Sham—SNI	− 16.687	0.000	− 16.936	0.000
Sham—Free-PRF	− 16.776	0.000	− 16.846	0.000
85VPRF—45VPRF	7.984	0.000	7.975	0.000
85VPRF—65VPRF	3.947	0.002	3.943	0.002
85VPRF—100VPRF	− 8.074	0.000	− 7.885	0.000
85VPRF—SNI	12.022	0.000	12.007	0.000
85VPRF—Free-PRF	12.111	0.000	11.918	0.000
65VPRF—45VPRF	4.037	0.001	4.032	0.001
65VPRF—100VPRF	− 4.127	0.001	− 3.943	0.002
65VPRF—SNI	8.074	0.000	8.065	0.000
65VPRF—Free-PRF	8.164	0.000	7.975	0.000
45VPRF—100VPRF	− 0.090	1.000	0.090	1.000
45VPRF—Free-PRF	4.127	0.001	3.943	0.002
45VPRF—SNI	4.037	0.001	4.032	0.001
100VPRF—SNI	3.947	0.002	4.122	0.001
100VPRF—Free-PRF	4.037	0.000	4.032	0.001
SNI—Free-PRF	− 0.090	1.000	0.090	1.000

Data were analyzed by Kruskal–Wallis test.

*PRF* pulsed radiofrequency, *Free-PRF* Free pulsed radiofrequency, *SNI* spared nerve injury.

**Figure 3:**
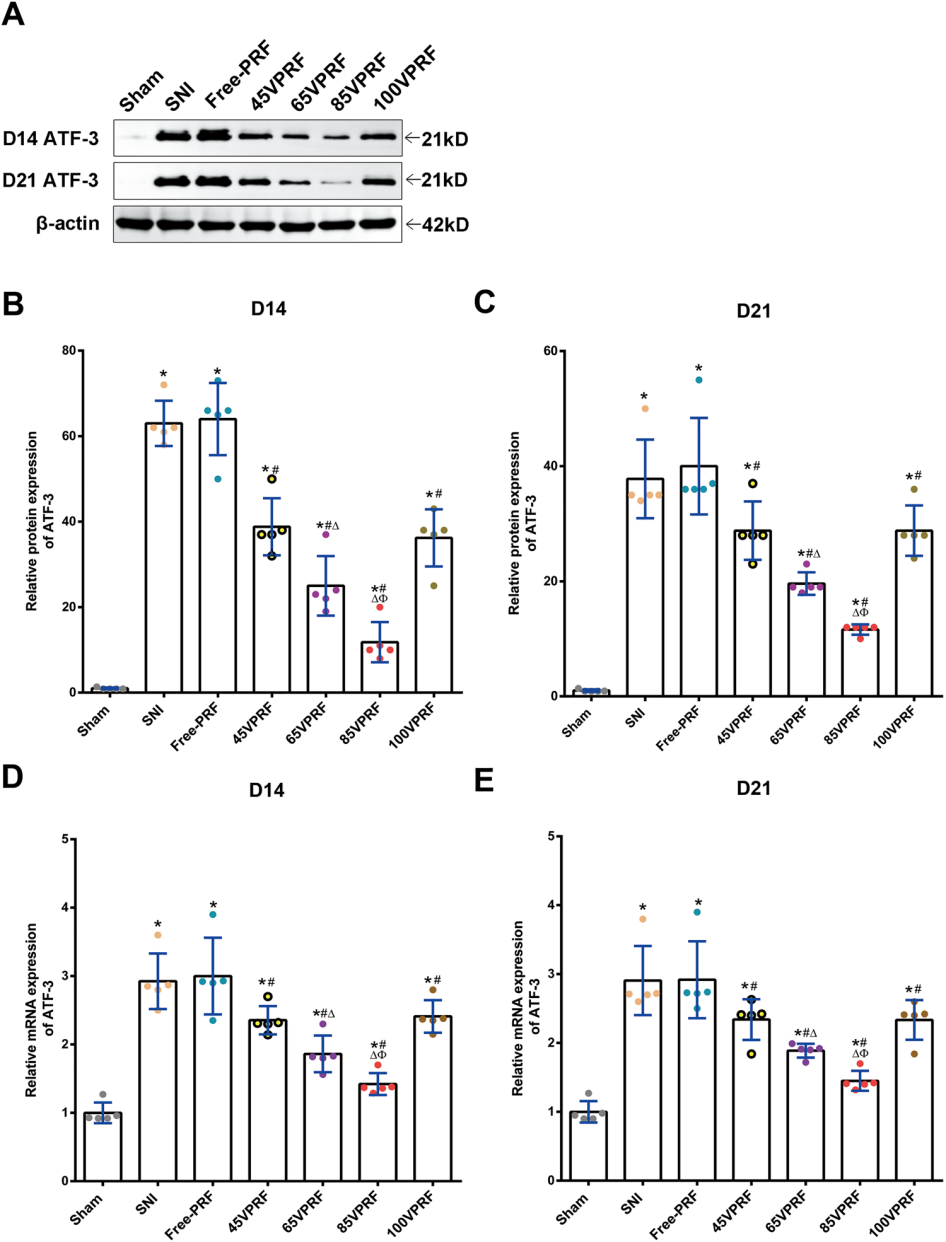
ATF-3 expression in the DRG on days 14 and 21 after SNI. **(A–C)** Western blot analysis of the levels of ATF-3 protein in the DRG on days 14 and 21 post-SNI. **(D,E)** Real-time PCR analysis of ATF-3 mRNA expression in the DRG on the 14th and 21st days post-SNI. Values represent the relative ratio of the ATF3 levels (normalized to β-actin) to the Sham group. Compared with those in the Sham group, ATF-3 levels in the other six groups were significantly increased on days 14 and 21 post-SNI. The ATF3 levels in the all PRF treatment groups were lower than those in the SNI group. No significant difference was found between the 45VPRF and 100VPRF groups. Moreover, the ATF3 levels in the 65VPRF group were lower than those in the 45VPRF and 100VPRF groups, but were significantly higher than those in the 85VPRF. Of all PRF treatment groups, the 85VPRF group had the lowest ATF-3 levels. One-way ANOVAs followed by the Tukey’s multiple comparisons test, n = 5 for each group. Data are expressed as the mean ± SD. *P < 0.05 vs. Sham; ^#^*P* < 0.05 vs. SNI; ^∆^*P* < 0.05 vs. 45VPRF; ^Φ^*P* < 0.05vs. 65VPRF.

Furthermore, we measured the ATF-3 expression in the L5 DRG after neuronal damage^21^. One-way ANOVA revealed a significant difference (Western blot analysis: *F* (6, 28) = 77.089, *P* < 0.001, *F* (6, 28) = 41.792, *P* < 0.001; Quantitative real-time PCR: *F* (6, 28) = 28.103, *P* < 0.001, *F* (6, 28) = 22.967, *P* < 0.001) in the protein and mRNA expression level of ATF-3 among groups on days 14 and 21 post-SNI. Compared with those in the Sham group, ATF-3 levels in the other six groups were significantly increased on days 14 and 21 post-SNI (all* P* < 0.05), which indicated that the remaining six groups of animals all had varying degrees of damage in the L5 DRG. The ATF3 levels in the all PRF treatment groups were lower than those in the SNI group (all *P* < 0.05). No significant difference was found between the 45VPRF and 100VPRF groups (*P* > 0.05). Moreover, the ATF3 levels in the 65VPRF group were lower than those in the 45VPRF and 100VPRF groups, but were significantly higher than those in the 85VPRF (all *P* < 0.05). Of all PRF treatment groups, the 85VPRF group had the lowest ATF-3 levels, suggesting that the nerve damage was repaired to varying degrees by different voltages of PRF, with the 85VPRF group showing the best improvement (all* P* < 0.05) (Fig. 3, Supplementary Table S2).

### HVPRF treatment resulted in a greater autophagy increase in microglia in the SCDH of SNI rats than in the SVPRF group

**Figure 4:**
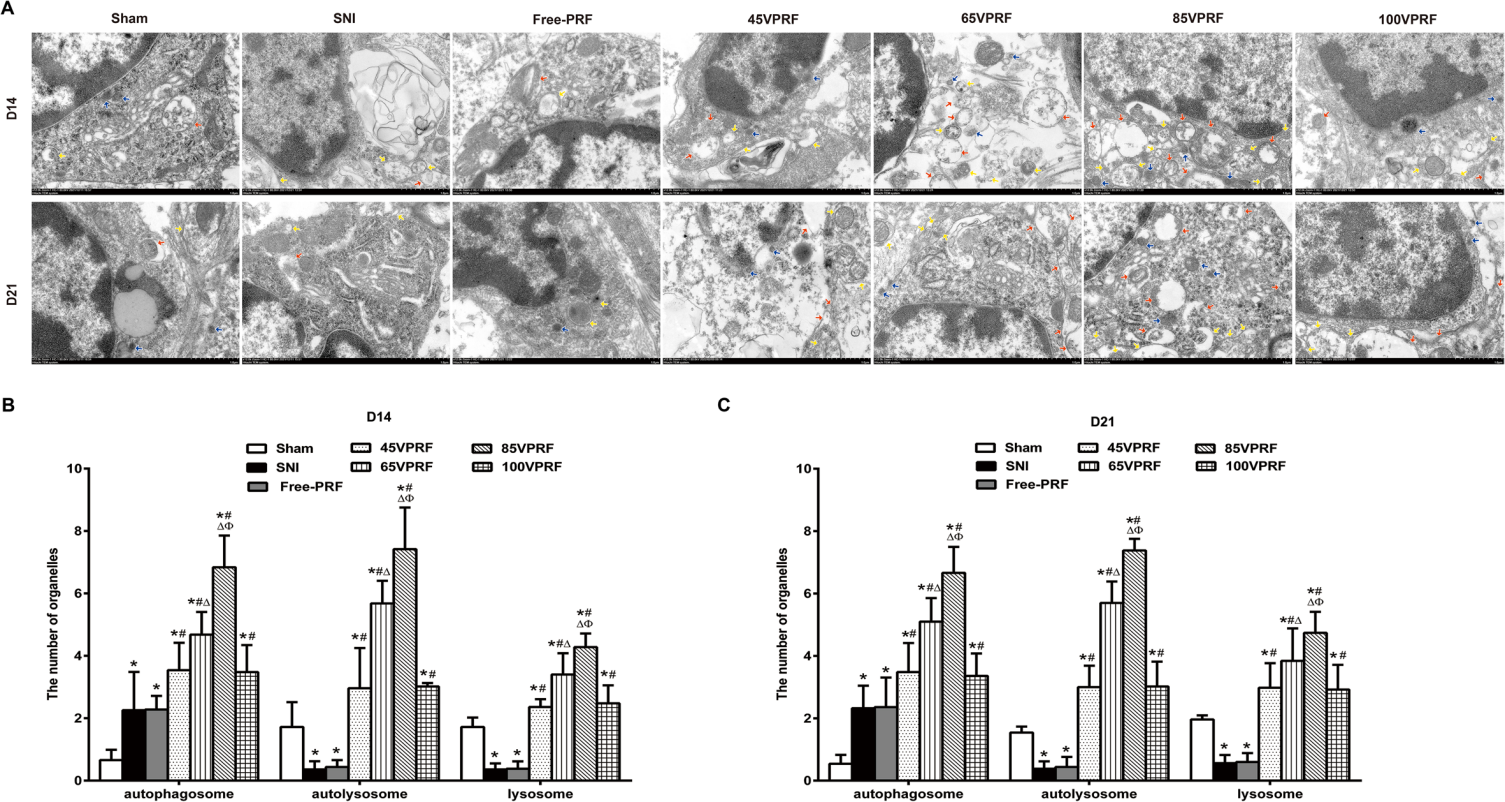
Effects of PRF on autophagy in the spinal dorsal horn by transmission electron microscopy. **(A)** Representative electron microscopy views of autophagosomes (yellow arrow), autolysosomes (red arrow) and lysosomes (blue arrow) in the spinal dorsal horn of the seven groups. Scale bar = 1 μm; magnification × 12,000. **(B,C)** The number of autophagosomes, autolysosomes and lysosomes on the 14th and 21st days of the seven groups in per vision was determined. In the Sham group, autophagosomes, lysosomes and autolysosomes in microglia in the SCDH of the rats were not commonly observed. The SNI and Free-PRF groups showed a slight elevation in the number of autophagosomes but fewer lysosomes and autolysosomes. Compared with those in the SNI group, more autophagosomes, lysosomes and autolysosomes were observed in microglia of 45VPRF, 65VPRF, 85VPRF and 100VPRF groups. The number of autophagosomes, lysosomes and autolysosomes in the 65VPRF group were higher than those in the 45VPRF and 100VPRF groups, but were significantly lower than those in the 85VPRF group. Of all PRF treatment groups, the 85VPRF group had the highest number of autophagosomes, lysosomes and autolysosomes. One-way ANOVAs followed by the Tukey’s multiple comparisons test, n = 5 for each group. Data are expressed as the mean ± SD. *P < 0.05 vs. Sham; ^#^*P* < 0.05 vs. SNI; ^∆^*P* < 0.05 vs. 45VPRF; ^Φ^*P* < 0.05vs. 65VPRF.

To determine whether the autophagic regulation of microglia in the SCDH is involved in the therapeutic mechanism of PRF, electron microscopy was used to investigate changes in the autophagic vesicles in microglia in the SCDH. In the Sham group, autophagosomes, lysosomes and autolysosomes in microglia in the SCDH of the rats were not commonly observed. The SNI and Free-PRF groups showed a slight elevation in the number of autophagosomes but fewer lysosomes and autolysosomes, which indicated impaired autophagy (*P* < 0.05). Compared with those in the SNI group, more autophagosomes, lysosomes and autolysosomes were observed in microglia of 45VPRF, 65VPRF, 85VPRF and 100VPRF groups, which indicated more activated autophagy and a more functional autophagy process^22^ (all *P* < 0.05). The number of autophagosomes, lysosomes and autolysosomes in the 65VPRF group were higher than those in the 45VPRF and 100VPRF groups (all *P* < 0.05), but were significantly lower than those in the 85VPRF group. Of all PRF treatment groups, the 85VPRF group had the highest number of autophagosomes, lysosomes and autolysosomes (all *P* < 0.05) (Fig. 4, Supplementary Table S2).

### HVPRF reversed the SNI-induced alterations in the expression of neuroinflammatory cytokines in the SCDH of rats

**Figure 5:**
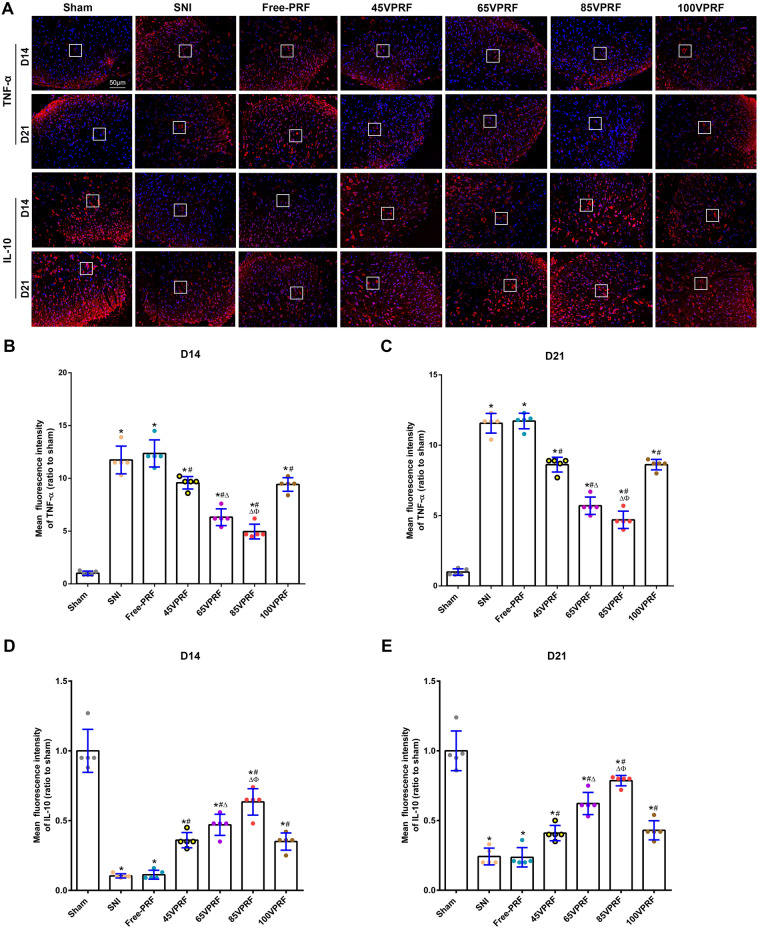
Representative images of TNF-α and IL-10 expression in spinal cord horns among the different groups by immunofluorescence. (**A**) Expression levels of TNF-α and IL-10 in spinal cord horns among different groups by immunofluorescence. The red fluorescence indicates TNF-α and IL-10 expression, while blue fluorescence indicates the nucleus. Scale bar = 50 µm; magnification × 200. **(B,C)** Analysis of TNF-α in the dorsal horn of the spinal cord in rats on days 14 and 21 post-SNI.** (D,E)** Analysis of IL-10 in the dorsal horn of the spinal cord in rats on days 14 and 21 post-SNI. The intensity of TNF-α immunofluorescence were higher, whereas IL-10 fluorescence intensity were lower in the SNI group than those in the Sham group on the 14th and 21st days post-SNI. Compared with those in the SNI group, the intensity of TNF-α immunofluorescence were decreased and the intensity of IL-10 immunofluorescence were increased in each PRF group. The changes in decreasing TNF-α and increasing IL-10 fluorescence intensity were more remarkable in the 65VPRF group than those in the 45VPRF and 100VPRF groups (all *P* < 0.05). In particular, of all PRF treatment groups, the 85VPRF group had the lowest TNF-α fluorescence intensity and highest IL-10 fluorescence intensity. One-way ANOVAs followed by the Tukey’s multiple comparisons test, n = 5 for each group. Data are expressed as the mean ± SD. *P < 0.05 vs. Sham; ^#^*P* < 0.05 vs. SNI; ^∆^*P* < 0.05 vs. 45VPRF; ^Φ^*P* < 0.05vs. 65VPRF.

**Figure 6:**
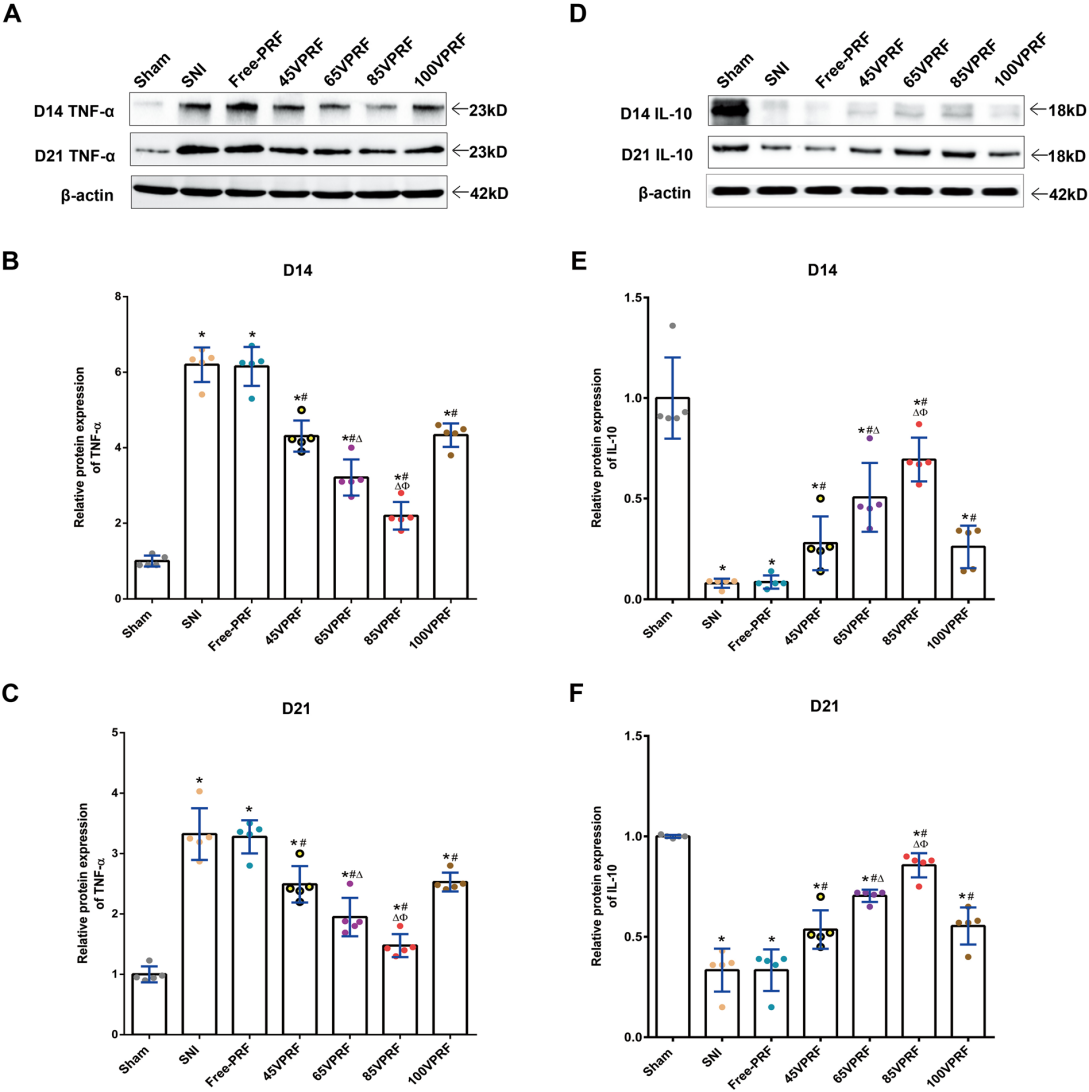
Different expression levels of TNF-α and IL-10 in the spinal dorsal horn among different groups on days 14 and 21 after SNI. **(A–C)** Western blot analysis of the levels of TNF-α protein in the DRG on days 14 and 21 post-SNI. **(D–F)** Western blot analysis of the levels of IL-10 protein in the DRG on days 14 and 21 post-SNI. Values represent the relative ratio of the TNF-α and IL-10 levels (normalized to β-actin) to the Sham group. The levels of TNF-α were higher, whereas IL-10 expression levels were lower in the SNI group than those in the Sham group on the 14th and 21st days post-SNI (all *P* < 0.05). Compared with those in the SNI group, the levels of TNF-α were decreased and the IL-10 levels were increased in each PRF group. The changes in decreasing TNF-α and increasing IL-10 levels were more remarkable in the 65VPRF group than those in the 45VPRF and 100VPRF groups. In particular, of all PRF treatment groups, the 85VPRF group had the lowest TNF-α levels and highest IL-10 levels. One-way ANOVAs followed by the Tukey’s multiple comparisons test, n = 5 for each group. Data are expressed as the mean ± SD. *P < 0.05 vs. Sham; ^#^*P* < 0.05 vs. SNI; ^∆^*P* < 0.05 vs. 45VPRF; ^Φ^*P* < 0.05vs. 65VPRF.

To clarify the efficacy of HVPRF on SNI-induced neuroinflammation, the expression of TNF-α and IL-10 in the SCDH of rats were measured by immunofluorescent histochemistry (Fig. 5, Supplementary Table S2) and Western blot (Fig. 6, Supplementary Table S2). Significant differences were observed in the relative protein expression of the proinflammatory cytokine TNF-α (Mean fluorescence intensity: *F* (6,28) = 108.107, *P* < 0.001, *F* (6,28) = 261.017, *P* < 0.001; Western blot: *F* (6,28) = 116.97, *P* < 0.001, *F* (6,28) = 50.668, *P* < 0.001) and the anti-inflammatory cytokine IL-10 (Mean fluorescence intensity: *F* (6,28) = 73.798, *P* < 0.001, *F* (6,28) = 64.635, *P* < 0.001; Western blot: *F* (6,28) = 36.044, *P* < 0.001; *F* (6,28) = 50.097, *P* < 0.001) among groups on the 14th and 21st days post-SNI. The levels of TNF-α were higher, whereas IL-10 expression levels were lower in the SNI group than those in the Sham group on the 14th and 21st days post-SNI (all *P* < 0.05). Compared with those in the SNI group, the levels of TNF-α were decreased and the IL-10 levels were increased in each PRF group (all *P* < 0.05). The changes in decreasing TNF-α and increasing IL-10 levels were more remarkable in the 65VPRF group than those in the 45VPRF and 100VPRF groups (all *P* < 0.05). In particular, Of all PRF treatment groups, the 85VPRF group had the lowest TNF-α levels and highest IL-10 levels.

## Discussion

NeP afflicts millions of people worldwide and is often accompanied by a deterioration of life quality^23^, and the increasing global challenges of patients with NeP urgently need to be addressed. Limited by the side effects and poor efficacy of the current treatments, more effective methods are needed. In recent years, HVPRF has been preliminarily verified in clinical practice to improve outcomes in NeP patients^11,20^. In the present study, we found that a single HVPRF application on the DRG provided a significant relief of mechanical allodynia, cold allodynia, and spontaneous pain induced by SNI in rats, and the therapeutic efficacy of 85VPRF was superior to that of PRF applied with other voltages. In addition, the neuropathic damage resulting from SNI could be partially mitigated at the microscopic level after PRF application on the DRG. Another major discovery was that PRF treatment modulated the autophagy process and the expression of inflammatory cytokines. HVPRF, especially 85VPRF, was more effective than SVPRF in enhancing microglial autophagy and increasing the levels of IL-10 in the SCDH, whereas it decreased the levels of TNF-α. These findings provide new mechanisms related to the analgesic efficacy of the HVPRF mode and support its clinical application in the treatment for NeP.

Although the application of HVPRF on DRG have received increasing attention, studies have shown that HVPRF is more effective than SVPRF. Luo et al.^7^ demonstrated that a higher output voltage and current field intensity increased the analgesic effect for trigeminal neuralgia. Several clinical studies have also shown that increasing the output voltage of PRF can significantly improve refractory neuralgia of the infraorbital nerve^24^, and postherpetic neuralgia^25^ without increasing the risk of side effects. However, the output voltage was manually adjusted to the maximum voltage (bearable without causing pain in conscious patients) in these studies, and the voltage values were inconsistent. In addition, the relationship between output voltage and efficacy, the most suitable output voltage, and the effect of different output voltages on the target tissue have not been further studied. In this study, outputs of 45 V, 65 V, 85 V and 100 V were used to simulate real clinical situations and explore the optimal therapeutic voltage. The results showed that the mechanical allodynia, cold allodynia, and spontaneous pain were observed on day 3 and peaked on day 7 post-SNI and remained stable for the subsequent observation period, which is in accordance with the results of a previous study^26^. The three pain behaviors started to improve on the 1st day after PRF and remained stable until the 14th day after treatment. In comparison, the three pain behaviors were relieved in the 65VPRF group at all time points after the 8th day of SNI, which were better than those in the 45VPRF and 100VPRF groups, but were significantly worse than those in the 85VPRF group. The three pain behaviors in the 65VPRF and 85VPRF groups were significantly better than those in the 45VPRF and 100VPRF groups, and that in the 85VPRF group showed the most significant improvement. These results indicated that 65 V and 85 V PRF applied to the DRG for NeP are more effective than the standard voltage (45 V). Hence, HVPRF can further enhance the efficacy. However, the efficacy of PRF in treating NeP decreased when the voltage was increased from 85 to 100 V in the present study, and the potential mechanisms remain to be studied in near future.

In this study, we observed the ultrastructure of DRG and the expression of ATF3 in each group to investigate whether there was any damage or repair effect of different voltages of PRF on the DRG and the trends in their changes. ATF3 is a protein that responds rapidly to stress and if cells are injured, ATF3 expression increases rapidly, and it is considered a biomarker of nerve injury^23^. Intriguingly, the RT-PCR and Western blot measurement of ATF3 levels in the DRG suggested that instead of exacerbating DRG nerve injury, different voltages of PRF could reduce the DRG injury induced by SNI. Besides, the observation of ultrastructure in the DRG showed that unlike those in the Sham group, the DRG neurons and nerve fibers in the SNI group were severely damaged indicating severe nerve injury. In addition, all PRF groups showed only partial myelin degeneration with irregular shape and segmental loose lamellar structure. These results showed that PRF with different voltage outputs does not aggravate nerve damage but promotes repair of the damaged nerve fibers to varying degrees, which not only confirms the safety of PRF but also elucidates its restorative effect, which is consistent with previously reported studies^13,27,28^. Tun et al.^28^ found that the damaged myelin axons in the PRF group only showed separation of myelin structures. The separation of the myelin structure can lead to the blockage or disruption of nerve signals through this neural pathway, which can lead to reversible neuronal inhibition. In addition, newly formed myelinated axons were also observed. The observed neural changes may be related to the interaction of electromagnetic fields with cell membranes. Podhajsky et al.^29^ showed that PRF treatment at 42 °C could cause transient endoneurial edema, ultrastructural axon damage, abnormal mitochondrial and membrane morphological changes, microtubule and microfilament collapse, and myelin destruction of DRG and sciatic nerve in rats. However, no side effects were observed in clinical practice, suggesting that the benefits of PRF's therapeutic effect may outweigh the minor nerve tissue damage in patients^30^. In the study, we observed that the neuronal damage in the 65VPRF and 85VPRF groups was less severe than that in the 45VPRF group. Most notably, 85VPRF exhibits greater efficacy in improving the ultrastructure of the DRG than 65VPRF, it is the preferred therapeutic voltage. However, the therapeutic effect of PRF on NeP decreased and the improvement of neural ultrastructure was worse than those of 85 V and 65 V when the voltage increased from 85 to 100 V. Cosman et al.^31^ found that high electric fields may disrupt neuronal membrane and function in liver and egg-white models. Cell membrane electroporation at high electric fields is destructive and irreversible. It causes a large increase in the permeability of the cell membrane and an imbalance of molecules within the cell, which can lead to cell stress or death. Electrolysis at low electric field intensity creates temporary pores, leading to Na^+^ and K^+^ conduction and membrane depolarization. This may be the reason why the effect of 100VPRF was reduced. These findings provide the theoretical basis for the better efficacy of the clinical application of HVPRF in the treatment of NeP, however, the mechanisms through which the high voltage fields promote nerve repair are still largely unknown.

Autophagy plays an essential role in maintaining the fine control of cell metabolism and the homeostasis of the internal environment^32,33^. Autophagy relies on lysosomes to degrade the damaged and redundant proteins and organelles. Recent studies have revealed that the impairment of autophagic flux is closely associated with the pathological processes of numerous neurodegenerative diseases^34^. Since neurons are nonregenerative cells, nonphysiological and abnormal autophagy activity may disrupt the dynamic balance of cell metabolism and organelle renewal, thus causing pathological changes in neural tissue structure^35^. Microglia are typical brain-resident macrophages, and selective clearance of damaged mitochondria by microglial autophagy is essential for neuronal survival and pain signaling^36^. It is known that central sensitization is recognized as a critical pathophysiological mechanism for NeP, in which the microglia within the SCDH play a role by regulating autophagy and neural inflammatory response. Moreover, autophagy has been associated with microglia-induced inflammation in NeuP^17^. The interactions between these processes and their role in the central nervous system have been a hotspot of research in recent years^37,38^. Specifically, nociceptive information acquired after peripheral nerve injury (PNI) is integrated by the DRG and transmitted into the SCDH, where it activates microglia to produce inflammatory substances, induces central sensitization, and promotes the development of NeP, but the activation of autophagy can inhibit this change^39,40^. Recently, accumulating studies revealed that the dysregulation of autophagy was observed in NeP, which contributed to mechanical allodynia by triggering neuroinflammation^41^. Hydrogen-rich saline inhibited microglial activation, inhibiting inflammation in an autophagy-mediated manner^17^. Recent study reported that TREM2-deficient microglia robustly induced autophagy via impairing mTOR signaling^42^. In a study of rats with left L5 spinal nerve ligation (SNL), rapamycin, which is an autophagy inducer, was found to improve NeP by activating autophagy and inhibiting interleukin-1β in the spinal cord of rats^43^. Another study showed that the inhibition of autophagy led to mechanical allodynia in an SNI-induced model, while modulation of autophagy relieved NeP by inhibiting microglia-mediated neuroinflammation^20^. Further research revealed that PRF applied to L5 DRG could improve SNL-induced NeP, which might have been partly due to the regulatory effects on neuronal autophagy in the SCDH^44^. These results are compatible with the results of the present study. In the SNI and Free-PRF groups, the endoplasmic reticulum of microglia was dilated, mitochondria were swollen and deformed with vacuolization, and bimodal structures of autophagosomes were observed in the cytoplasm. The number of autophagosomes was significantly higher in the 65VPRF and 85VPRF groups than that in the 45VPRF group, and the increase in the number of autophagosomes in the 85VPRF group was significantly different from that in the 65VPRF group. Thus, DRG intervention with different voltages of PRF enhanced microglial autophagy in the SCDH in SNI rats and effectively improved NeP.

A common mechanism underlying NeP is neuroinflammation. Tissue injury and inflammation can release a variety of inflammatory mediators, whose main functions are pro-inflammatory and anti-inflammatory. The common pro-inflammatory factors include TNF-α, IL-1β, IL-6 and interferon, etc., and the common anti-inflammatory factors include IL-10, IL-4 and tumor necrosis factor binding protein^45^. Previous studies have shown that pro-inflammatory factors and anti-inflammatory factors co-regulate NeP^46^.

As an inflammatory factor, TNF-α is involved in the production and maintenance of NeP by regulating upstream and downstream channels and cytokines. Gruber-Schoffnegger et al.^47^ found that intrathecal injection of exogenous TNF-α could lead to thermal and mechanical hyperalgesia in rats. In the SNL rat model, it was found that the expression of TNF-α was up-regulated in the DRG tissue of the L4-L5 spinal cord segment, and intrathecal injection of TNF-α inhibitors could alleviate the mechanotactile pain and thermal hyperalgesia of rats^48^. When exogenous TNF-α was injected into the DRG of the injured nerve roots in the chronic CCI model, it was transported to the injured site and enters the SCDH^49^, causing allodynia in the receptive fields of the ligated and non-ligated nerves^50^. In recent years, it has been reported that the expression of TNF-α in sciatic nerve, DRG and spinal dorsal horn of animal models of NeP with different types of PRF treatment is significantly inhibited while the pain behavior is improved^51^. The secretion of TNF-α may be an important hub between the immune system, NeP, and PRF treatment.

The mechanism by which IL-10 participates in NeP is thought to be through inhibiting the release of pro-inflammatory factors^46^. Krukowski et al.^52^ found that the increase of IL-10 was conducive to reducing NeP caused by chemotherapy. Due to the short half-life of IL-10 in cerebrospinal fluid, direct intrathecal injection of IL-10 recombinant protein can only temporarily reverse NeP caused by PNI^53^. However, intrathecal injection of a plasmid encoding IL-10 protein can reverse pain hypersensitivity for a long time^54^, and so do intramural polymer-mediated IL-10^53^. Shen et al.^55^ showed that IL-10 not only inhibits Nav1.8 sodium current in the DRG, but also reverses TNF-α-induced up-regulation of Nav1.8 sodium channels, thereby alleviating NeP. It is suggested that IL-10 may regulate NeP by inhibiting TNF-α and related pro-inflammatory factors.

In various models of NeP, key cytokines including TNF-α and IL-10, released by activated microglia are thought to cooperate in regulating neuroinflammation in the spinal cord^56,57^. Therefore, we measured the levels of TNF-α and IL-10 in the spinal cord. The results indicated that TNF-α levels increased and IL-10 levels decreased with the development of NeP, but after PRF intervention, IL-10 levels increased and TNF-α levels decreased in the four PRF groups, which was accompanied by a rebound in the three pain behaviors. In particular, the reversal was most obvious in the 85VPRF group. Based on these results, the therapeutic effect of HVPRF can be attributed to its regulatory role in inflammatory mediators, such as TNF-α and IL-10. The relationship between autophagy and inflammation are complex. Previous studies showed that autophagy negatively regulates inflammation to prevent the harmful amplification of inflammatory factors^58^. This is consistent with our research.

The present study has some limitations. First, the therapeutic effect of HVPRF was only tested on the 14th and 21st days after SNI, and the overall trend of microglial autophagy levels in the SCDH was not observed, so the long-term efficacy of HVPRF needs further study. Second, intra-SCDH injection of chloroquine, which is an autophagy inhibitor, or rapamycin was not performed to further confirm. Third, the conclusions of the study are based on the results of an animal model, and it is not known whether consistent results could be obtained in human studies. These deficiencies will be addressed in future in-depth research.

## Conclusion

**Figure 7:**
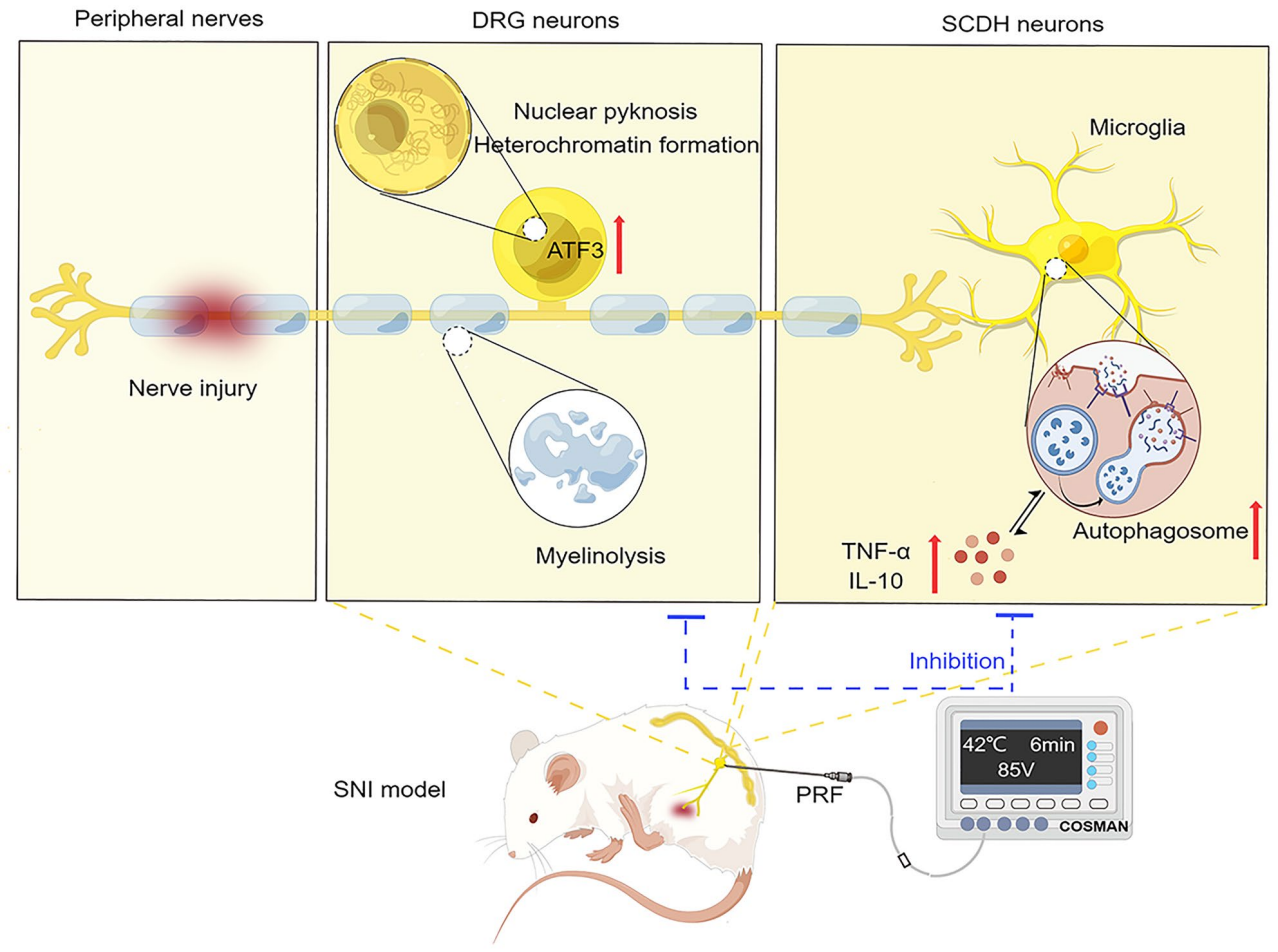
Schematic diagram of the analgesic mechanism of PRF on DRG in neuropathic pain by Figdraw. [Red arrows represent upregulation. Blue arrows indicate deregulation. The ATF3 and ultrastructure in dorsal root ganglion (DRG) neurons and the autophagy level of spinal cord dorsal horn (SCDH) are involved in neuropathic pain].

In conclusion, HVPRF achieved better pain relief than SVPRF, and 85VPRF exhibited the best therapeutic efficacy. The underlying mechanisms may be related to repairing the nerve damage and improving the DRG ultrastructure while regulating spinal microglial autophagy and thereby alleviating neuroinflammation (Fig. 7).

## Materials and methods

### Animals

**Figure 8:**
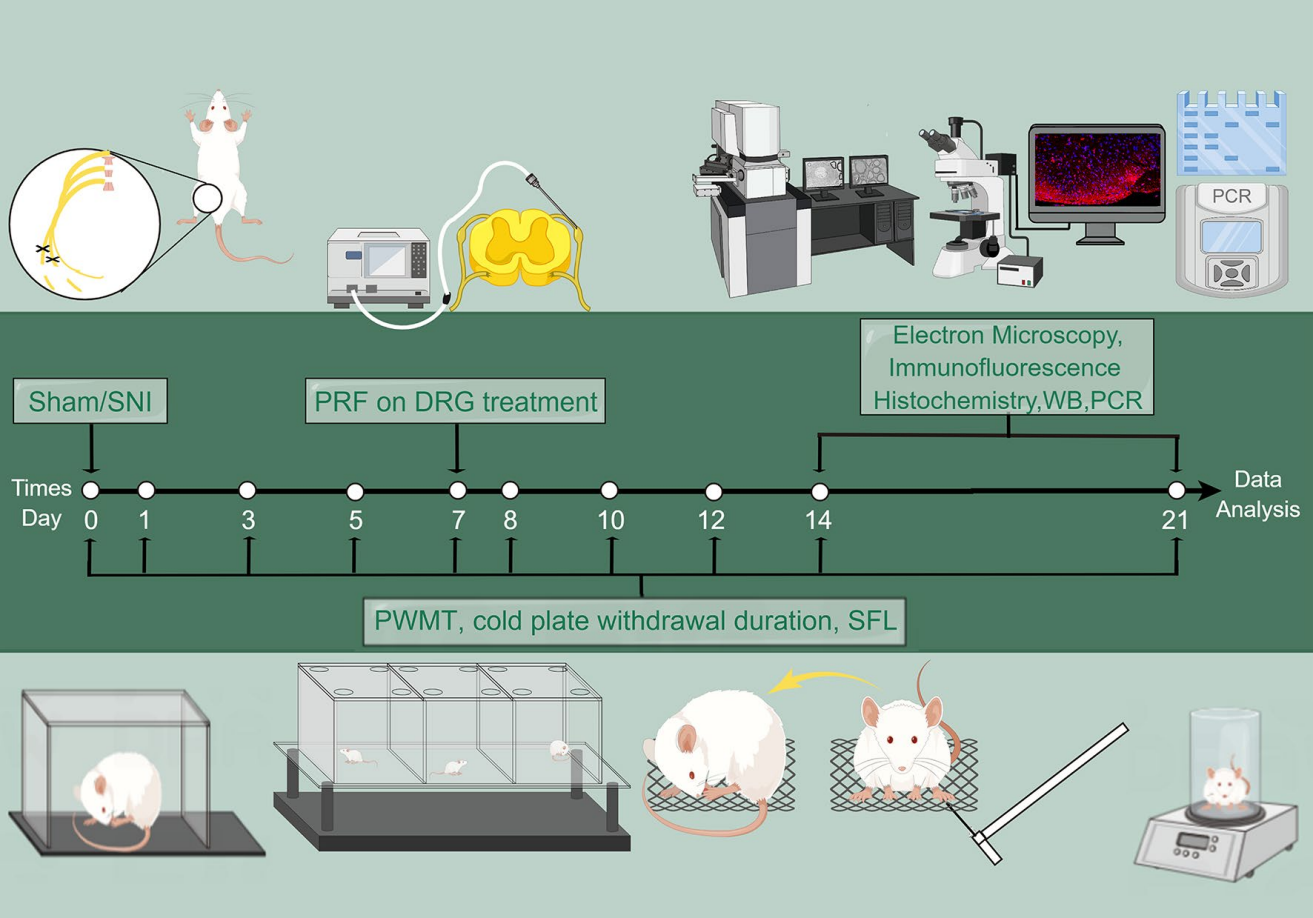
Schematic representation of the experimental schedule. PRF was applied to the left DRG of rats. Sensory function was tested by assessing the mechanical allodynia (paw mechanical withdrawal threshold, PMWT), cold allodynia (withdrawal duration of cold plate test), and spontaneous pain (spontaneous foot lifting, SFL) at different time points (0, 1, 3, 5, 7, 8, 10, 12, 14 and 21 days). The samples were taken and tested on the 14th and 21st days after SNI.

A total of 280 male Sprague‒Dawley rats weighing 250–300 g were provided by the Animal Center of Fujian Medical University (No. SCXK (Min) 2016-0002). The rats were maintained with a 12-h light/dark cycle under a controlled temperature of 22 ± 2 °C and constant humidity. Rats had free access to food and water until 24 h before SNI. All procedures were approved by the Experimental Animal Welfare Ethics Committee of Fujian Medical University (No: FJMU IACUC 2022-0635) and complied with guidelines from the Committee for Research and Ethical Issues of the International Association for the Study of Pain, and Animal Research Reporting of In Vivo Experiments. To date, the output voltage used in clinical radiofrequency devices is typically set to less than 100 V, and voltages over 60 V are considered high-voltage^25,59^. In the present study, all rats were randomly assigned to seven groups (n = 40/group): Sham group received sham operation; SNI group received SNI; Free-PRF group received SNI and Free-PRF; 45VPRF group, 65VPRF group, 85VPRF group and 100VPRF group received SNI and PRF treatment with corresponding voltage. All rats were tested for the PMWT, cold allodynia and SFL before surgery and at 1, 3, 5, 7, 8, 10, 12, 14, and 21 days post-SNI. On days 14 and 21 post-SNI, 20 rats in each group were euthanized at each time point, and the spinal cord and DRG were collected for follow-up tests. The experimental procedure is presented in Fig. 8.

### The SNI model

In accordance with the previous description^60^, the SNI procedure was carried out. In brief, rats were anesthetized intraperitoneally with 1% sodium pentobarbital (50 mg/kg) to expose the three terminal branches of the left sciatic nerve. Transection of the common peroneal and tibial nerves was performed, leaving the sural nerve intact. In the Sham group, the nerves were exposed without being cut or ligated.

### Treatment

Rats were treated with PRF under anesthesia on day 7 after SNI. The laminectomy and facetectomy were performed to expose the left L5 DRG in both the Free-PRF and PRF groups. The radiofrequency (RF) electrode (type 20 G, 5 cm long, 4 mm active tip) was placed close to the L5 DRG by direct visualization using an RF device (Cosman RFG-4 Generator). The parameter settings are as follows^61^: pulse rate of 2 Hz, voltage of 45 V/65 V/85 V/100 V for the corresponding group, maximum temperature of 42 °C, pulse width of 20 ms, and stimulation time of 6 min. Free-PRF was conducted by placing electrode on the DRG, but no simultaneous PRF was delivered.

### Behavioral tests

All behavioral tests were performed by observers who were unaware of the specific surgical procedures, interventions, and eventual differences in treatment outcomes.

### PMWT

The PMWT was tested before SNI and on the 1st, 3rd, 5th, 7th, 8th, 10th, 12th, 14th and 21st postoperative days. After a minimum of 1 h of habituation in the test room, the rats were placed in individual Plexiglas cells (10 × 15 × 20 cm) on a wire mesh floor for 15 min. Using the up-and-down method as previously described, Von Frey filaments (bending force of 1.0, 1.4, 2.0, 4.0, 6.0, 8.0, 10.0, and 15.0; Stoelting, Wood Dale, IL, USA) were applied to the lateral plantar surface (the sural-innervated territory) of the left hindpaw^62^. The filament was subjected to the bending force for 3–4 s applied to the hind paw plantar. Starting with one filament and a bending force of 2 g, the next lighter filament was treated if a paw withdrawal response was observed. The next stronger bending force filament was applied if the paw withdrawal response was not observed. The series of von Frey filaments were with bending force ranging from 1 to 15 g. Avoiding further contact with the filament, quickly turning the head away, scratching the stimulated area, or attacking the filament were considered positive responses. The PMWT was measured according to the methods reported by Chaplan et al.^62^. An allodynic rat was defined as the one where PMWT is < 4.0 g (withdrawal in response to non-noxious tactile stimulus).

### Cold allodynia

An ice box was filled with crushed ice cubes and water, and a metal aluminum plate was placed on the surface of the box. The temperature of the plate was controlled at 4–6 °C. The test was performed after modification of the previously described method^63^. After a minimum of 1 h of habituation in the test room, the rats were placed on the surface of an aluminum plate under a glass cover with a ventilation hole (20 × 15 × 20 cm). The rats were acclimated for 5 min, and the total time that the hind paw was lifted from the cold plate was recorded for 5 min. Foot lifting associated with exploratory behavior, locomotion and body repositioning was excluded.

### SFL

Spontaneous pain was assessed by evaluating SFL. For this test, each rat was placed on a plate kept at a neutral temperature (30 ± 1 °C), and after 1 h of acclimatization, the number of times that the animal rapidly (< 0.5 s from beginning to maximum elevation) lifted the ipsilateral hind paw and showed aversive behavior (e.g., foot shaking/licking) was measured for 5 min. Foot lifting associated with exploratory behavior, locomotion and body repositioning was excluded^64^.

### Transmission electron microscopy

The left L5 DRG and spinal cord of rat was extracted on day 14 or 21 after SNI. The samples were fixed in 2% glutaraldehyde, washed 3 times in phosphate buffer (pH 7.4), postfixed in 1% osmium tetroxide in phosphate buffer for 2 h at 25 °C, and dehydrated in alcohol with increasing concentrations. Subsequently, tissues were embedded in epoxy resin, cut into semithin slices about 2 μm, stained with azure methylene blue, positioned, cut into ultrathin slices approximately 60 nm, and stained with osmic acid. Observation of DRG neuron cell bodies and myelin morphology was made by a transmission electron microscope (HT7700, Hitachi, Hitachi, Japan). As reported previously^65^, nerve fiber myelin is graded based on its ultrastructural appearance (Table 3). The quantitative assessment included 250 (the spinal cords of 5 rats were taken from each group, and 50 myelinated axons were observed in each spinal section, which was equivalent to 5 × 50 = 250 axons in each group) myelinated axons in each group. Grades 0 and 1 were considered better grades in the statistical analysis. The preparation of transmission electron microscopy sections of the left SCDH was performed as described above. Ten fields of view were randomly selected in each case, and the number of autophagic organelles in the SCDH was recorded for statistical analysis.

**Table 3:** Ultrastructural grading system of myelinated axons.

Score	Category
Grade 0	Normal
Grade 1	Separation in myelin configuration
Grade 2	Interruption in myelin configuration
Grade 3	Honeycomb appearance
Grade 4	Collapsed myelin-forming ovoids

### Immunofluorescence histochemistry

The rats were perfused with 200 mL phosphate buffer (pH 7.4) containing 4% paraformaldehyde and then with 200 mL saline. Then, the left L5 DRG was removed and fixed in 4% paraformaldehyde for 1 day. Subsequently, the tissues were allowed to equilibrate in 30% sucrose in phosphate-buffered saline (PBS) overnight at 4 °C. Tissue was collected in 0.01 M PBS (pH 7.4) after cutting the left L5 DRG using a cryostat. The tissue was penetrated with 0.3% Triton X-100 and processed with primary antibodies for rabbit anti-rat TNF-α (ab6671, 1:100, Abcam. RRID:AB_305641) and IL-10 (ab34843, 1:100, Abcam. RRID:AB_733110) overnight at 4 °C. The sections were then incubated with CY3-conjugated secondary antibody (AP132C, 1:100, Sigma, RRID:AB_92489) for 1 h at room temperature. Then, fluorescence mounting medium with 4′,6-diamidino-2-phenylindole was finally added. The sections were observed under a Nikon fluorescence microscope. The average fluorescence intensity was quantified to assess the expression level of TNF-α and IL-10 using ImageJ software (National Institutes of Health Inc.).

### Western blot analysis

The left L5 DRG and SCDH were collected and frozen at − 80 °C after the rats were sacrificed on the 14th or 21st day after SNI. Tissue samples were lysed in RIPA buffer containing protease and phosphatase inhibitors (Beyotime). The concentration of protein in the lysates was determined by a bicinchoninic acid protein assay kit (Beyotime). The proteins were transferred to polyvinylidene fluoride membranes after separation by electrophoresis (Millipore), and the membranes were then blocked with 5% nonfat milk in TBST, followed by incubation with primary antibodies: ATF-3 (ab216569, 1:1000, Abcam. RRID:AB_3073865), TNF-α (ab6671, 1:1000, Abcam. RRID:AB_305641), IL-10 (ab34843, 1:1000, Abcam. RRID:AB_733110) and β-actin (#4967, 1:1000, Cell Signaling Technology) at 4 °C overnight and with horseradish peroxidase-conjugated secondary antibody (#31466, 1:1000, Thermo Scientific) at room temperature for 1 h. The signals of the target proteins and β-actin were tested by a chemiluminescence detection system (Bio-Rad Laboratories Inc.). The expression level of relative protein was normalized by using β-actin as an internal reference, and the intensity of band was analyzed by ImageJ software^66^.

### Quantitative real-time PCR

A TRIzol Reagent (Servicebio) was used to isolate total RNA from tissues. A Primer Script RT reagent kit (Servicebio Co. Ltd., Wuhan, China) was used to translate the RNA into cDNA, and qPCR was performed on a Bio-Rad real-time PCR system using 2xSYBR Green PCR Master Mix (Servicebio Co. Ltd., Wuhan, China). The primers utilized for ATF3 were as follows: (sense) 5′-GGGTCACTGGTGTTTGAGGATT-3′ and (antisense) 5′-TTTGTTTCTTTCCCGCCG-3′. The primers used for GAPDH were as follows: (sense) 5′-CTGGAGAAACCTGCCAAGTATG-3′and (antisense) 5′-GGTGGAAGAATGGGAGTTGCT-3′. GAPDH was used as the internal control. The gene expression was calculated using the 2^−ΔΔCT^ method.

### Statistical analysis

Statistical analyses were completed using SPSS 21.0 and GraphPad Prism 9 software. All measurement data were tested for normality (Kolmogorow–Smironov test) and equal variance (Levene test). All measurement data conformed to normal distribution and homogeneity of variance. The measurement data are presented as the mean ± standard deviation (SD). Behavioral data were analyzed using two-way repeated measures analysis of variance (ANOVA) and Bonferroni post hoc tests for pairwise multiple comparisons. Data of autophagic organelles number, ATF3 levels and inflammatory cytokine levels were analyzed by ANOVA followed by Tukey’s multiple comparisons test. Data of myelin sheath damage grade was analyzed by Kruskal‒Wallis test. Differences were considered statistically significant when *P* < 0.05.

### Ethical approval

All experiments were approved by the Experimental Animal Welfare Ethics Committee of Fujian Medical University (No: FJMU IACUC 2022-0635) and complied with guidelines from the Committee for Research and Ethical Issues of the International Association for the Study of Pain. The study is reported in accordance with ARRIVE guidelines.

### Supplementary Information

Supplementary Information 1.

Supplementary Information 2.

Supplementary Information 3.

Supplementary Information 4.

Supplementary Information 5.

Supplementary Information 6.

Supplementary Information 7.

Supplementary Table S1.

Supplementary Table S2.

Supplementary Information 8.

Supplementary Information 9.

## Data Availability

The datasets generated during and/or analysed during the current study are available from the corresponding author on reasonable request.
