# Supplementary figures and images for: High-voltage pulsed radiofrequency improves ultrastructure of DRG and enhances spinal microglial autophagy to ameliorate neuropathic pain induced by SNI
Source: Sci Rep. 2024 Feb 24;14:4497. doi: 10.1038/s41598-024-55095-5 (PMC10894304; doi:10.1038/s41598-024-55095-5)

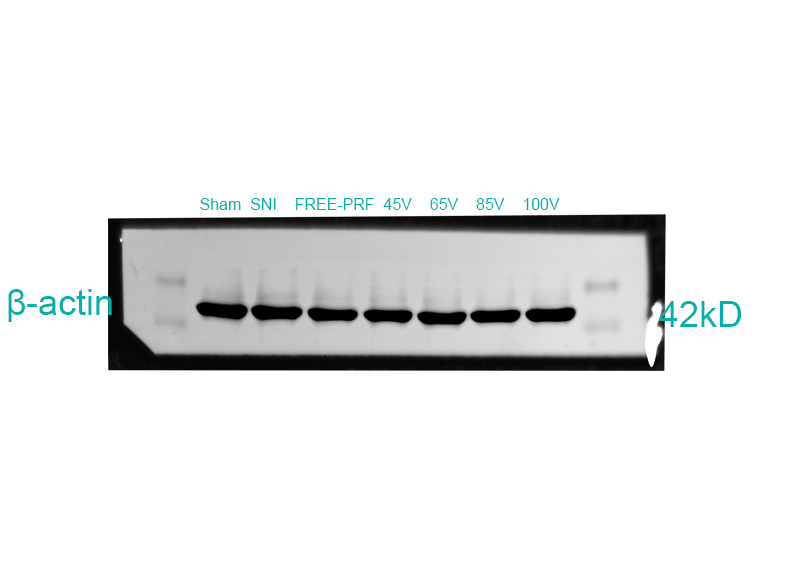

Supplement: Supplementary file 1 — Supplementary Information 1. [file 41598_2024_55095_MOESM1_ESM.jpg]

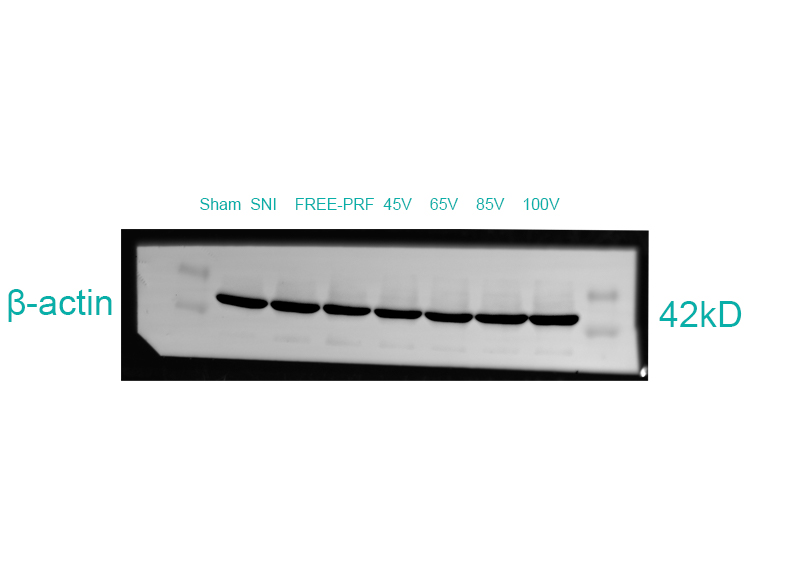

Supplement: Supplementary file 2 — Supplementary Information 2. [file 41598_2024_55095_MOESM2_ESM.jpg]

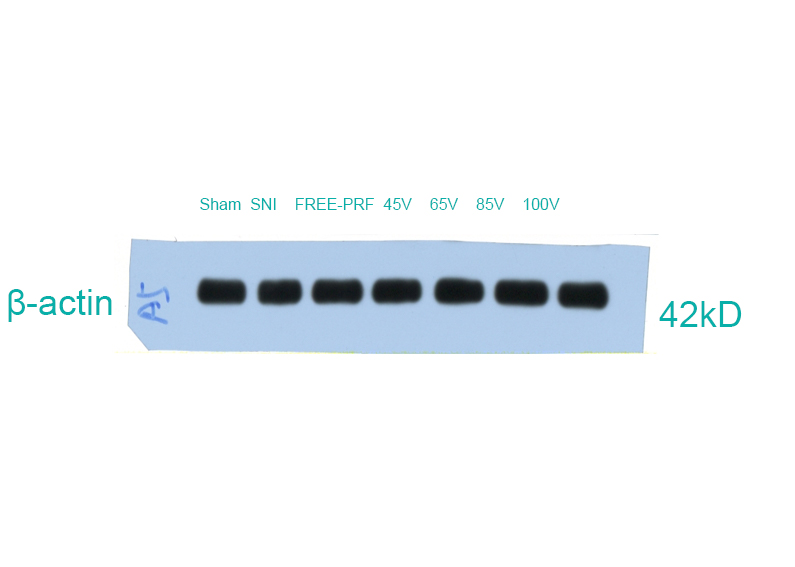

Supplement: Supplementary file 3 — Supplementary Information 3. [file 41598_2024_55095_MOESM3_ESM.jpg]

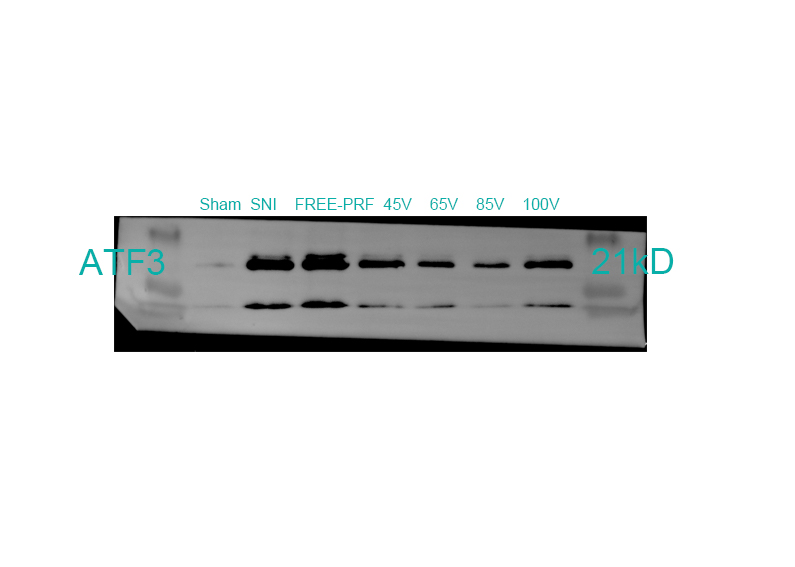

Supplement: Supplementary file 4 — Supplementary Information 4. [file 41598_2024_55095_MOESM4_ESM.jpg]

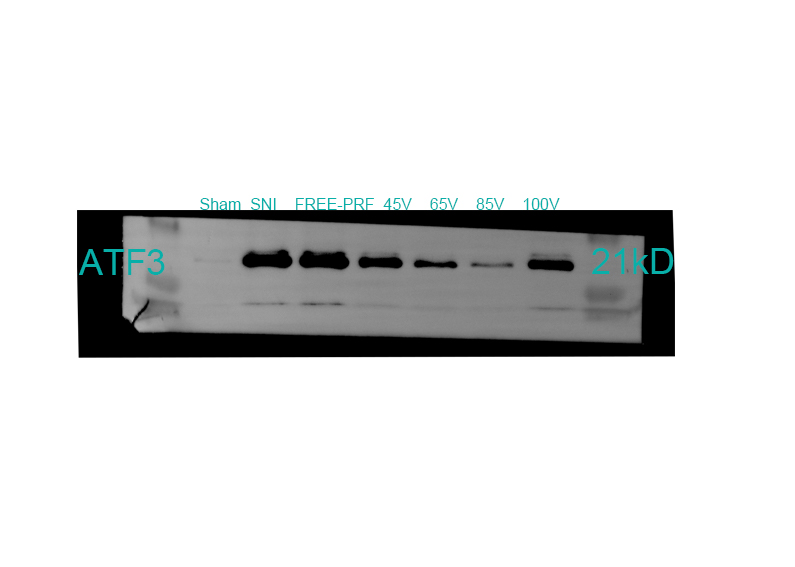

Supplement: Supplementary file 5 — Supplementary Information 5. [file 41598_2024_55095_MOESM5_ESM.jpg]

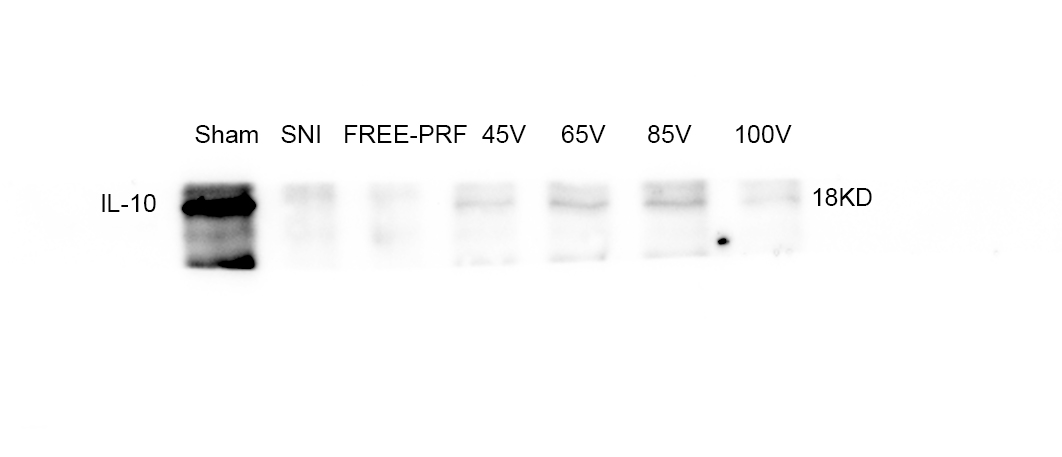

Supplement: Supplementary file 6 — Supplementary Information 6. [file 41598_2024_55095_MOESM6_ESM.jpg]

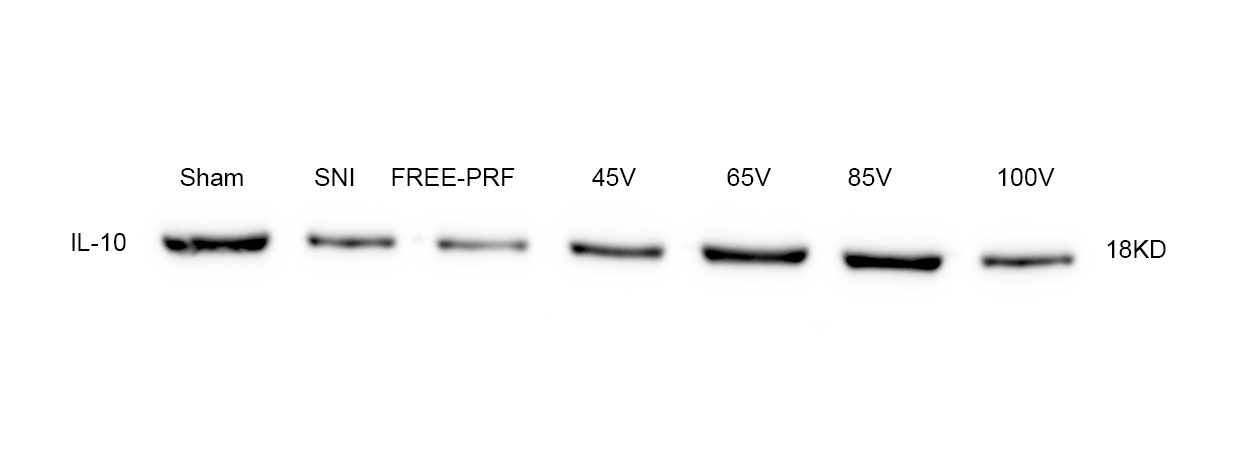

Supplement: Supplementary file 7 — Supplementary Information 7. [file 41598_2024_55095_MOESM7_ESM.jpg]

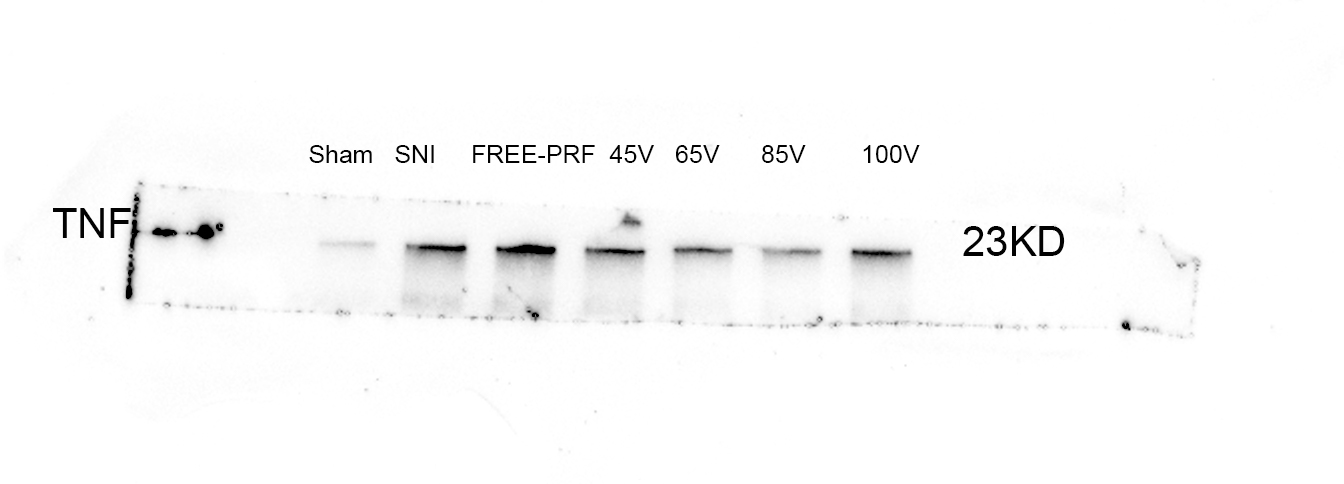

Supplement: Supplementary file 10 — Supplementary Information 8. [file 41598_2024_55095_MOESM10_ESM.jpg]

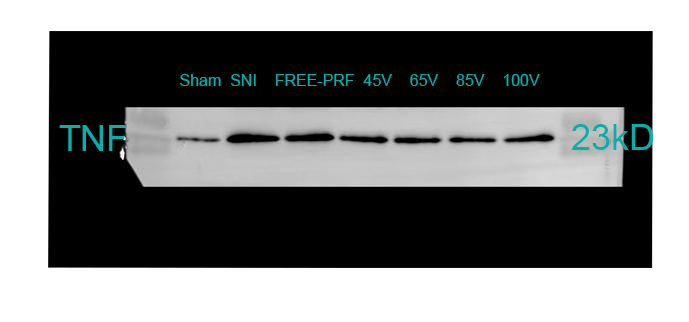

Supplement: Supplementary file 11 — Supplementary Information 9. [file 41598_2024_55095_MOESM11_ESM.jpg]
